# miR193b Promotes Apoptosis of Gastric Cancer Cells via Directly Mediating the Akt Pathway

**DOI:** 10.1155/2020/2863236

**Published:** 2020-05-25

**Authors:** Ruyue Tian, Hailun Jiang, Linlin Shao, Yang Yu, Qingdong Guo, Bangwei Cao, Shuilong Guo

**Affiliations:** ^1^Department of Oncology, Beijing Friendship Hospital, Capital Medical University, Beijing 100050, China; ^2^Institute of Medicinal Biotechnology, Chinese Academy of Medical Sciences and Peking Union Medical College, Beijing 100050, China; ^3^Department of Gastroenterology, Beijing Friendship Hospital, Capital Medical University, National Clinical Research Center for Digestive Disease, Beijing Digestive Disease Center, Beijing Key Laboratory for Precancerous Lesion of Digestive Disease, Beijing 100050, China

## Abstract

Gastric cancer (GC) is one of the most common and fatal malignancies worldwide. MicroRNAs (miRNAs) play a critical role in tumor initiation, proliferation, and metastasis of gastric cancer. miR193b has been identified as a tumor suppressor in a variety of tumor types; however, its role in gastric cancer is yet to be determined. Here, we found a significant downregulation of miR193b expression in both human gastric cancer tissues (*p* < 0.05) and human gastric cancer cell lines (*p* < 0.01). Furthermore, the expression level of miR193b correlated with the tumor type, tumor size, and clinical stage (*p* < 0.05). In vitro, miR193b overexpression inhibited cell survival and induced apoptosis in GC cell lines, indicating that miR193b plays a role in the development of gastric cancer. KRAS was verified as the target of miR193b, and KRAS overexpression attenuated miR193b-induced apoptosis (*p* < 0.05). Moreover, we found that the Akt pathway negatively regulated miR193b, also affecting apoptosis. Further analyses indicated that PIK3CA mutation and KRAS amplification are two mutually exclusive pathways (*p* < 0.01), and we hypothesize that both two pathways could result in the carcinogenic overactivation of KRAS. Thus, our results suggest that the Akt-miR193b-KRAS axis may act as a mechanism affecting apoptosis in gastric cancer cells.

## 1. Introduction

Gastric cancer (GC) is the fifth most frequently occurring malignancy and ranks as the third leading cause of cancer-related deaths worldwide [1]. Although there has been considerable progress in uncovering the genetic alterations driving tumor initiation and growth in this condition, the molecular mechanisms underlying GC progression require further investigation [2]. Exploring this issue will help in identifying novel diagnostic and therapeutic targets for human gastric cancer.

MicroRNAs (miRNAs) are short, noncoding, single-stranded RNAs, which predominantly interact with genes by binding to the 3′ untranslated region (UTR) of their target mRNAs, thus inhibiting mRNA translation [3, 4]. miRNAs play a significant role in the regulation of many key biological processes, including tumor formation and progression [5–8]. In gastric cancer, miRNAs have been shown to target several apoptosis-related genes, such as Mcl-1 (miR512-5p), bcl-2 (miR34), MIF (miR451), and EGR2 (miR150). Besides, miRNA deregulation can promote cell cycle progression, migration, and invasion, by altering the levels or translation of their target mRNA [9].

Recently, it was shown that miR193b is frequently downregulated in several tumor types, including esophageal cancer, pancreatic cancer, and ovarian cancer [10–12]. Previous work demonstrated that miR193b acts as a tumor suppressor in the hematopoietic system and that it can induce apoptosis and G1/S-phase arrest in various human Acute Myelocytic Leukemia (AML) subgroups [13]. Nevertheless, whether miR193b regulates apoptosis in GC cells, the molecular mechanisms underlying this regulation, and the upstream pathways involved in miR193b signaling is a topic that remains largely unexplored.

Akt signaling plays a crucial role in tumor progression. Mutations in the key components of the Akt pathway, such as PI3KCA, p53, PTEN, and FOXO1, are often observed in gastric cancer. This could induce an abnormal activation of the Akt signaling pathway and contribute to the development and progression of GC [14–17]. Our previous study found that Akt signaling could promote gastric cancer cell proliferation through suppression of miR365 expression [17]. Whether other Akt-regulated miRNAs affect gastric cancer progression deserves further exploration.

Here, we showed that miR193b expression was downregulated in human GC and that it was associated with certain clinical characteristics. Further experiments illustrated that apoptosis of GC cells was regulated through the Akt-miR193b-KRAS axis.

## 2. Materials and Methods

### 2.1. Human Gastric Tissues

Samples from 50 GC patients were obtained from the Beijing Friendship Hospital of China. All patient samples included paired gastric cancer and adjacent normal samples. All tissue samples were stored in liquid nitrogen prior to RNA extraction. Patient characteristics, including sex, age, tumor type, tumor size, and clinical stage, are showed in Table 1. This experiment was authorized by the Ethics Committee of Beijing Friendship Hospital, Capital Medical University. miR193b expression profiles and clinical stage of GC patients were obtained from the TCGA data portal (https://tcga-data.nci.nih.gov/tcga/).

### 2.2. Cell Culture and Transfection

A panel of gastric cancer cell lines, GES-1, AGS, SNU-16, MGC-803, BGC-823, and SGC-7901, were cultured in Dulbecco's modified Eagle medium supplemented with 10% fetal bovine serum (GIBCO) at 37°C and 5% CO_2_. All miRNAs, including miR193b mimics, miR193b inhibitor, and negative control mimics, were synthesized by GenePharma (Shanghai, China). miRNA transfections were performed using a Lipofectamine 3000 transfection reagent (Invitrogen). The KRAS expression vector was constructed by inserting human KRAS cDNA into the pCMV-HA vector. The KRAS vector, as well as Myr-Akt1 (Millipore), was transfected at a final concentration of 1 *μ*g/mL. The Akt inhibitor LY294002 was used with a final concentration of 100 nM, while IGF-1 was 100 ng/mL.

### 2.3. RNA Extraction and Quantitative Real-Time PCR

All experimental equipment was treated with DEPC water to ensure RNase-Free before RNA extraction. Instead of using the whole tissue, we cut the frozen tissue into appropriate sizes (about 30 mg) on ice. The tissue block (about 30 mg) and 1 ml TRIzol (Invitrogen) were placed in a homogenizer and homogenized on ice for 1 minute; then, the instructions to the next step were followed. The extracted RNA was quantified using a NanoDrop spectrophotometer. TaqMan MicroRNA Expression assays (Thermo Fisher Scientific Inc.) and QuantStudio™ 7 Flex System (Applied Biosystems Inc., Foster City, CA, USA) were used for miRNA quantification. PCR data was analyzed using the 2-*ΔΔ*Ct method, with U6 as an endogenous control. The following primers were used: U6 forward 5′-CTC GCT TCG GCA GCA CA-3′ and reverse 5′-AAC GCT TCA CGA ATT TGC GT-3′; miR193b forward 5′-AAC TGG CCC TCA AAG TCC CGC T-3′ and reverse 5′-CGC GAC TTT GAG GGC CAG TTT T-3′.

### 2.4. Cell Survival

Cell proliferation was detected using a MTS assay (SGC-7901, BGC-823, SGA). For the MTS assay, 3000 GC cells were seeded in each well of a 96-well plate. 72 hours after transfection, 20 *μ*L of the CellTiter 96 AQueous One Solution Reagent (Promega) was added into each well and cells were incubated at 37°C for 2 h. Following incubation, 490 nm absorbance was measured using an enzyme-labelled meter (SpectraMax M3, Molecular Devices).

### 2.5. Cell Apoptosis

For cell apoptosis analysis, cells were stained with Annexin V-FITC and 7-aminoactinomycinD (7-AAD) (BD Biosciences) for 15 min 48 hours after transfection and subsequently analyzed using a flow cytometer (BD FACSVerse).

### 2.6. Comet Assay

The comet assay was performed using the Alkaline Comet Assay Kit (Trevigen) according to the standard protocol. SYBR® Gold is recommended for DNA visualization by epifluorescence microscopy (OLYMPUS, FLUOVIEW, FV1200).

### 2.7. Dual-Luciferase Assay

The 3′UTR of the human KRAS mRNA was synthesized and cloned into the XhoI and NotI sites of the psi-CHECK-2 vector (Promega). miR193b was cotransfected with the psi-CHECK-2-KRAS-3′-UTR-WT or psi-CHECK-2-KRAS-3′-UTR-MUT1/2/3 vector into HEK293 cells. After incubation for 48 hours after transfection, cells were measured using the Dual-Luciferase Reporter Assay System (Promega). Position 303-309 of KRAS 3′UTR was mutated in MUT1; Position 1074-1080 of KRAS 3′UTR was mutated in MUT2; both two positions were mutated in MUT3.

### 2.8. Western Blotting

Total protein was extracted from cells after transfection, and protein concentration was measured using the BCA protein assay kit and quantified using a NanoDrop 2000c spectrophotometer (Thermo Scientific, Waltham, MA, USA). 40 *μ*g of each protein sample was separated using SDS-PAGE (10% sodium dodecyl sulfate polyacrylamide gel electrophoresis) and transferred to PVDF membranes (Millipore). GAPDH was used as an internal control. The following primary antibodies were used: Akt (Proteintech Group), p-Akt (Ser473) (Proteintech Group), cleaved-caspase3 (Proteintech Group), bcl-2 (Proteintech Group), bax (Proteintech Group), Rictor (Santa Cruz Biotechnology), and KRAS (Santa Cruz Biotechnology). The phosphorylation level of Akt in gastric cancer cells was expressed as the ratio of phosphorylated Akt to total Akt expression (p-Akt to Akt).

### 2.9. Statistical Analysis

GraphPad Prism software (version 5.0) was used for all statistical analysis. All values are expressed as means ± SD. Groups were compared using Student's *t*-test, and the results were considered statistically significant at *p* < 0.05.

## 3. Results

### 3.1. The Expression of miR193b Is Downregulated in Human GC Tissue Samples, and miR193b Levels Are Correlated with Clinicopathological Features of Human Gastric Cancer

**Figure 1:**
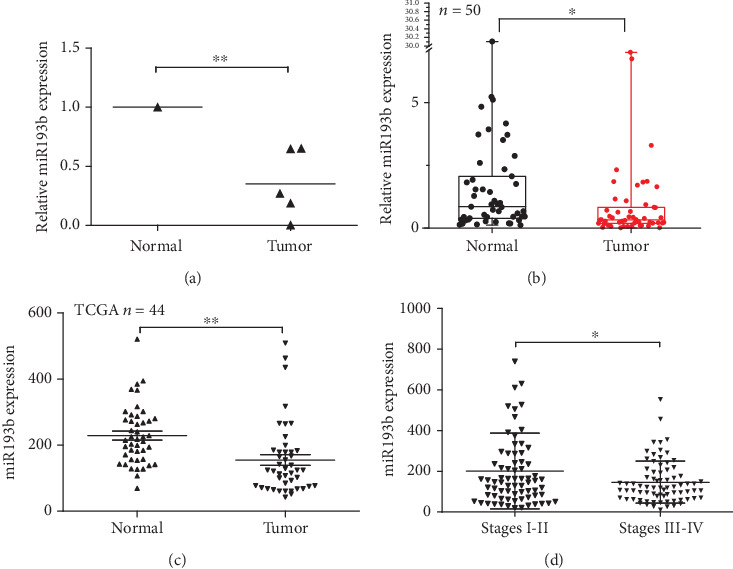
miR193b acts as a tumor suppressor by promoting apoptosis of GC cells. (a) miR193b levels in the normal epithelial cell line GES-1 and the gastric cancer cell lines AGS, N87, SNU-16, BGC-823, and SGC-7901. (b) The relative expression of miR193b in gastric normal tissues and matching cancer tissues (*n* = 50). Data represent the median flanked by the 25th and 75th percentiles. (c) miR193b expression of 44 pairs of GC and normal tissues (original data were extracted from TCGA GC dataset). (d) Patients with high TNM stage show lower miR193b expression (original data were extracted from TCGA GC dataset). ^∗^*p* < 0.05 and ^∗∗^*p* < 0.01 were calculated using Student's *t*-test.

Using Quantitative Real-Time PCR, we found that miR193b expression was markedly lower in 5 different GC cell lines (AGS, N87, SNU-16, BGC-823, and SGC-7901) than in the normal epithelial cell line GES-1 (Figure 1(a), *p* < 0.01). Moreover, across 50 human GC tissues, miR193b expression was significantly lower in the GC tissue compared to the matched adjacent normal tissue (Figure 1(b), *p* < 0.05). We further analyzed the TCGA database and found that miR193b expression was significantly lower in the GC tissue which was consistent with our experimental results (Figure 1(c), *p* < 0.01). Additionally, we revealed that low miR193b expression was associated with the advanced stage of GC (Figure 1(d), *p* < 0.05). To investigate the relationship between miR193b expression levels and clinicopathological features seen in gastric cancer patients, we divided 50 clinical cases into two groups: the downregulated group (*n* = 28) in which miR193b expression in tumors was less than or equal to 50% of the expression of adjacent normal tissues and the upregulated group (*n* = 22) in which miR193b expression in tumors was higher than 50% of the expression of adjacent normal tissues. As shown in Table 1, decreased miR193b expression was significantly correlated with signet-ring cell carcinoma (SRCC), associated with larger tumor size (The tumor volume of two patients could not be calculated due to incomplete collection of case information.) and advanced stage, suggesting that downregulation of miR193b may contribute to human gastric cancer progression (Table 1).

### 3.2. miR193b Enhances/Promotes Apoptosis of GC Cells

**Figure 2:**
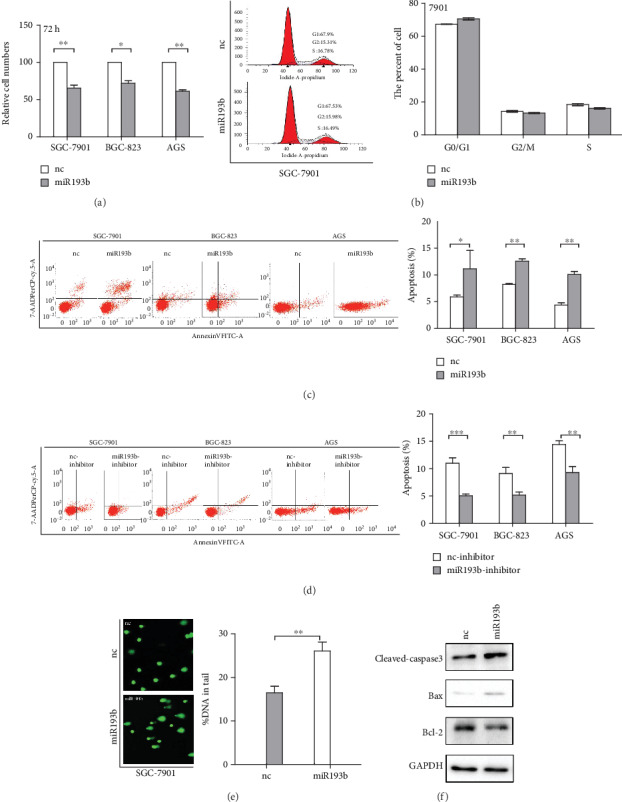
Effects of miR193b transfection on gastric cancer cell lines. (a) MTS assay showing cell viability. miR193b inhibits cell proliferation of SGC-7901, BGC-823, and AGS cells. Cell viability was determined by cell count 72 hours after transfection. (b) No difference in cell cycle progression was detected after transfection with miR193b. (c, d) Transfection with miR193b can induce apoptosis in GC cells (SGC-7901, BGC-823, and AGS) while miR193b inhibitor treatment reduced apoptosis compared to the negative control group. (e) Comet assay confirmed that overexpression of miR193b promotes DNA damage. (f) The protein levels of cleaved-caspase3, bax, and bcl-2 were determined by Western blot analysis after transfection with miR193b or negative-control miRNA. ^∗^*p* < 0.05 and ^∗∗^*p* < 0.01 were calculated using Student's *t*-test.

We next investigated the effect of miR193b on gastric cancer growth by overexpressing miR193b in GC cell lines. The MTS assay revealed that miR193b overexpression significantly inhibited gastric cancer cell growth in SGC-7901, BGC-823, and AGS cells (Figure 2(a)). FACS was used to detect cell apoptosis and cell cycle in GC cells. No difference in cell cycle progression was detected (Figure 2(b)), but a higher percentage of apoptotic cells were found in SGC-7901, BGC-823, and AGS cells transfected with miR193b. Conversely, miR193b-inhibitor treatment led to a lower percentage of apoptotic cells in GC cells (Figures 2(c) and 2(d)), since apoptosis could be triggered by DNA damage [18, 19], and previous studies have shown that miRNAs play an important role in the regulation of DNA damage and that the extent of DNA damage is correlated with apoptosis [20, 21]. We performed a comet assay to detect DNA damage in cells. miR193b-transfected cells exhibited significantly more DNA damage, as indicated by a larger and longer DNA tail and increased tail DNA% (*p* < 0.05; Figure 2(e)). Furthermore, to confirm the effect of miR193b on apoptosis induction, we used Western blot to detect the expression of apoptosis pathway-related proteins such as cleaved-caspase3, bax, and bcl-2. Cleaved-caspase3 and bax protein levels were upregulated after miR193b overexpression, while bcl-2 was downregulated (Figure 2(f)). Taken together, these results confirmed that miR193b induced apoptosis in GC cells. This induction of apoptosis may be mediated by the mitochondrial apoptotic pathway, as indicated by the changes in caspase3 activity, bax, and bcl-2.

### 3.3. KRAS Is a Target of miR193b in Gastric Cancer Cells

**Figure 3:**
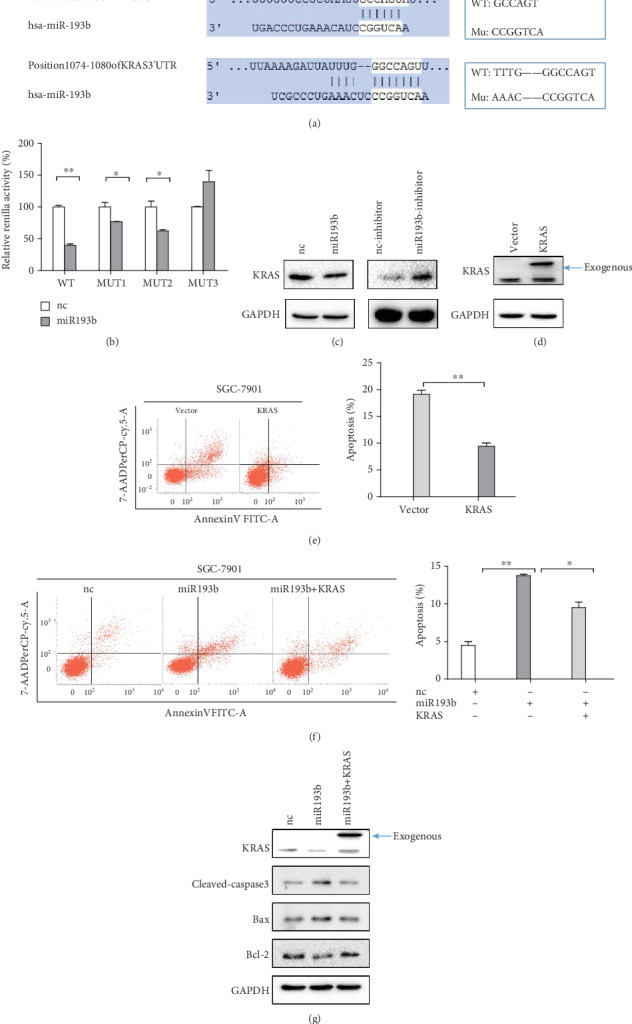
miR193b enhances apoptosis of GC cells by targeting KRAS. (a) miR193b has two binding sites in the 3′UTR of the KRAS mRNA. (b) Wild-type or mutant versions of the KRAS 3′UTR were cloned into the psiCHECK-2 vector. Renilla activities were measured in SGC-7901 cells cotransfected with miR193b and the KRAS-luciferase reporter. Position 303-309 of KRAS 3′UTR was mutated in MUT1; Position 1074-1080 of KRAS 3′UTR was mutated in MUT2; both two positions were mutated in MUT3. (c) KRAS protein expression decreased in SGC-7901 cells 48 hours after transfection with miR-193b. KRAS levels increased after transfection with miR193b-inhibitor. (d) Validation of the modified KRAS plasmid by Western blot assay. (e) Overexpression of KRAS reduces the apoptosis of SGC-7901 cells. (f) KRAS reduced miR193b-induced apoptosis in SGC-7901 cells. (g) Western blot analysis of KRAS, cleaved-caspase3, bax, and bcl-2 protein levels 48 hours after transfection with negative-control miRNA or miR193b or cotransfection with miR193b and KRAS. ^∗^*p* < 0.05 and ^∗∗^*p* < 0.01 were calculated using Student's *t*-test.

We next searched for targets of miR193b using the miRDB and TargetScan databases and identified KRAS as a potential miR193b target (Figure 3(a)). To determine whether KRAS was directly regulated by miR193b, we constructed luciferase reporters containing either a wild type or a mutant version of the KRAS 3′UTR. Cotransfection of miR193b mimics with psiCHECK-2-KRAS-WT-3′UTR led to significant reduction in renilla activity (Figure 3(b)). In contrast, mutation of the two miR193b binding sites (psiCHECK-2-KRAS-MUT3-3′UTR) did not show a reduction in renilla activity when cotransfected with miR193b mimics. Additionally, KRAS protein levels decreased in cells which overexpressed miR193b, while cells transfected with miR193b inhibitor showed an increase in KRAS expression, as detected by Western blot (Figure 3(c)). These results indicate that miR193b can directly regulate KRAS expression. After validation of the modified KRAS plasmid by the Western blot assay, we found that overexpression of KRAS reduces the apoptosis of SGC-7901 cells (Figures 3(d) and 3(e)). To determine the role of KRAS in miR193b-mediated apoptosis, we performed a rescue experiment. The result showed that overexpression of KRAS repressed miR193b-induced apoptosis (Figure 3(f)). Additionally, cells cotransfected with KRAS and miR193b showed that KRAS could rescue changes in apoptosis-related protein expression caused by miR193b overexpression (Figure 3(g)). These results suggested that miR193b induced apoptosis of SGC-7901 cells through negative regulation of KRAS expression.

### 3.4. miR193b Is Negatively Regulated by Akt Activity

**Figure 4:**
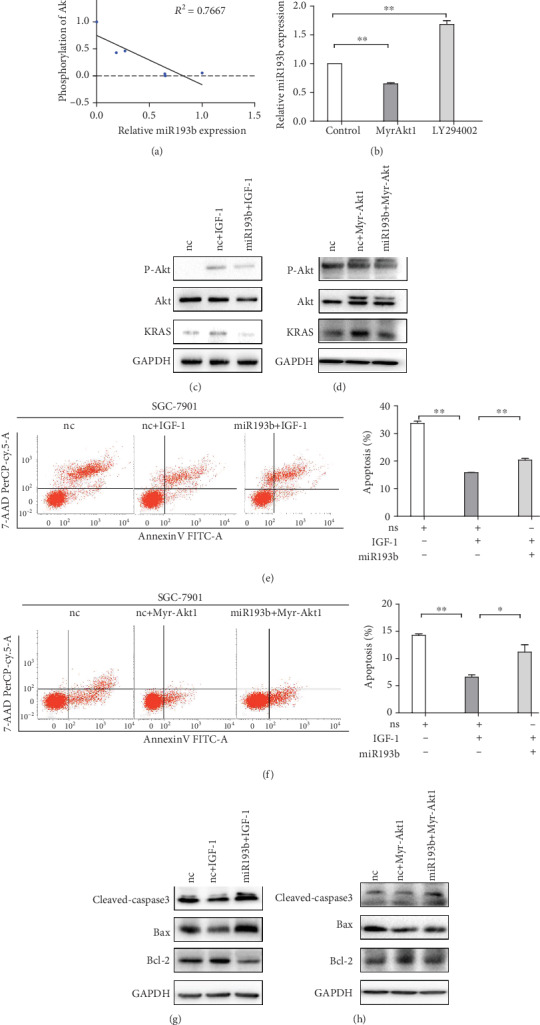
P-Akt is an upstream regulator of miR193b. (a) The expression of miR-193b was negatively correlated with the phosphorylation of Akt in gastric cancer cells. (b) qRT-PCR measurements of miR193b expression in cells transfected with Myr-Akt1 or treated with Akt inhibitor (LY294002). (c, d) Western blot measurements of KRAS protein after treatment with IGF-1 or Myr-Akt1. (e, f) miR193b reduced the inhibitory effect of IGF-1/Myr-Akt1 on apoptosis of SGC-7901 cells. (g, h) Western blot measurement of cleaved-caspase3, bax, and bcl-2 in cells overexpressing miR193b after IGF-1/Myr-Akt1 treatment. ^∗^*p* < 0.05 and ^∗∗^*p* < 0.01 were calculated using Student's *t*-test.

Using qRT-PCR, we assayed the expression levels of miR193b in GC cells. We find that the expression level of miR193b is negatively correlated with the phosphorylation of Akt which was confirmed by Western blotting (Figure 4(a)). The expression of miR-193b was detected in GC cell lines after transfection with myristoylated Akt1 (Myr-Akt1), a constitutively active form of Akt, or treatment with Akt phosphorylation inhibitor LY294002. We found that cells transfected with Myr-Akt1 exhibited reduced miR193b expression, while inactivation of Akt by LY294002 leads to increase in miR193b levels (Figure 4(b)). We used Western blot with p-Akt antibodies to detect the level of Akt activation in cells treated with IGF-1 or the constitutively active Myr-Akt1 and miR193b. Cells treated with both IGF-1/Myr-Akt1 and the miR193b mimic showed decreased p-Akt levels compared to cells treated with IGF-1/Myr-Akt1 and miRNA negative control. KRAS expression also followed the same trend as p-Akt, with less KRAS expressed in the cells treated with both IGF-1/Myr-Akt1 and miR193b (Figures 4(c) and 4(d)). To further confirm this finding, we performed a rescue experiment with IGF-1 or Myr-Akt1 alone or in combination with miR193b and assayed their effect on apoptosis. Cells treated with IGF-1/Myr-Akt1 showed decreased levels of apoptosis, while cotreatment with miR193b reduced this effect (Figures 4(e) and 4(f)). After treatment with IGF-1 or Myr-Akt1, Western blot analysis showed that cleaved-caspase3 and bax protein levels were downregulated while bcl-2 protein levels were upregulated, and cotreatment with miR193b could rescue these changes (Figures 4(g) and 4(h)). Transfection with the constitutively active Myr-Akt1 resulted in the same effects as IGF-1 treatment, further validating the role of the Akt activity in miR193b signaling. These results indicated that miR193b was negatively regulated by p-Akt.

### 3.5. Akt-miR193b-KRAS Axis Serves as a Mechanism Underlying Apoptosis of Gastric Cancer Cells

**Figure 5:**
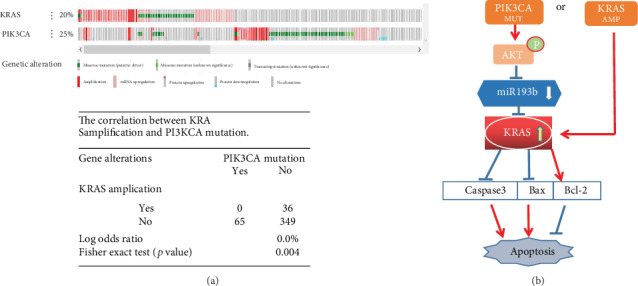
(a) KRAS amplification and PI3KCA mutation show mutual exclusion. (b) Schematic diagram of the pAkt-miR193b-KRAS pathway. ^∗^*p* < 0.05 and ^∗∗^*p* < 0.01 were calculated using Student's *t*-test.

There was mutual exclusion between KRAS amplification and PI3KCA mutation (Figure 5(a)). A schematic figure showed that both PIK3CA mutation and KRAS amplification play a carcinogenic role in GC by increasing the expression level of KRAS (Figure 5(b)).

## 4. Discussion

It has been demonstrated that miR193b has a suppressive function during cancer development in various tumors. For example, in the hematopoietic system, miR193b was reported as a cancer suppressor in AML by targeting essential molecules of the MAPK signaling pathway and thus controlling cell viability and proliferation [13]. In solid tumors, the dysregulation of miR193b may be caused by promoter methylation. miR193b can further interact with lncRNAs and other pivotal signaling pathways to inhibit cancer progression [22–24]. However, another study indicated miR193b as a cancer oncogene by targeting Smad3 in human glioma [25]. These data show that miR193b and the related mechanisms are still elusive and need to be addressed by further, in-depth research.

Some preliminary studies proposed that miR193b may be applied as a potential prognostic marker for gastric cancer [22, 26]. In the present work, we verified that miR193b expression was significantly reduced in gastric cell lines and tumor tissues, which was associated with tumor type, size, and TNM stage. miR193b expression was also verified through the TCGA database in our bioinformatic analysis, which made our experimental results more robust. Furthermore, we demonstrated that miR193b can decrease viability and induce apoptosis of GC cells. The changes in DNA damage highlight the proapoptotic function of miR193b. Taken together, these data suggest miR193b as a suppressor of gastric cancer progression.

In addition, it is noteworthy that our previous studies showed that miR193b can induce apoptosis in esophageal cancer cells and validated KRAS as its target [27]. KRAS upregulation is frequently observed in GC, and this could contribute to its tumorigenesis. These data suggested that miR193b-KRAS may play an important role in gastric cancer as well. We identified KRAS as a target of miR193b and found evidence that it could be negatively regulated by this miRNA in gastric cancer. Indeed, KRAS overexpression attenuated miR193b-induced apoptosis and the changes in apoptosis-related proteins. Therefore, our study sheds new light on these mechanisms and shows that miR193b induces apoptosis of GC cells by targeting KRAS.

Earlier research had shown that the Akt pathway regulates various miRNAs involved in tumor occurrence and development [28, 29]. In particular, our previous study indicated that the Akt signaling pathway promoted GC development by inhibiting miR365 expression, which is located in the same cluster as miR193b [17]. Our present results indicate that miR193b is negatively regulated by the Akt pathway. Indeed, the activation of the Akt signaling pathway through IGF-1 or Myr-Akt1 increased the expression of KRAS, whereas miR193b expression counteracted this effect. Further experiments indicated that the overexpression of miR193b altered the levels of apoptosis and apoptosis-related proteins induced by the activation of the Akt pathway. Our data indicate that miR193b mediates the effect of the Akt pathway on apoptosis through downregulation of KRAS expression, which is the first reported mechanism of miR193b in GC.

PIK3CA is a key protooncogene in the PI3K-Akt signaling pathway. The mutation of PIK3CA is a carcinogenic activator of the Akt pathway, which could promote cell proliferation and tumor growth [30]. In our data analysis from the TCGA database [31, 32], we found that PIK3CA has a mutation rate of 22.49% and that it ranks in the top 20 of all mutated genes in gastric cancer. KRAS amplification is seen in as many as 13% of GCs. Interestingly, we found that there was no PIK3CA mutation in patients exhibiting KRAS amplification. Further analysis of the data suggested that there was mutual exclusion between KRAS amplification and PI3KCA mutation. KRAS can be activated by reducing miR193b expression, which, in turn, is caused by Akt phosphorylation after PI3KCA mutation or by KRAS amplification itself. KRAS activation could change the expression of apoptosis-related proteins, further influencing cell apoptosis. Therefore, we hypothesize that two mutually exclusive pathways exist, resulting in carcinogenic overactivation of KRAS. Since the oncogenic RAS proteins cannot be targeted with drugs [33], the Akt-miR193b-KRAS axis shows promise for new therapeutic approaches.

Collectively, we found that miR193b showed lower expression in GC tissues and correlated with the clinicopathological features of the tumor. As a tumor suppressor in gastric cancer, miR193b is negatively regulated by the activated Akt pathway and could decrease cell viability, inducing apoptosis, by targeting KRAS. Due to the mutual exclusion between KRAS amplification and PI3KCA mutation, we suggest that both events play a carcinogenic role in GC by increasing the expression level of KRAS. These results indicate that the Akt-miR193b-KRAS axis might serve as a promising diagnostic biomarker and potential therapeutic target for gastric cancer. However, this study has some limitations. For example, the clinical role of miR-193b in vivo and miRNA measurements in whole tissue need further investigation. Moreover, further analyses and larger sample sizes will be necessary to validate these results.

## Figures and Tables

**Table 1:** The correlation between miR193b expression and clinicopathological features in human gastric cancer.

Factors	miR193b expression		*p* value
	Downregulated	Upregulated	
Age (year)			
≥60	18	13	
<60	10	9	0.707
Sex			
Male	22	16	
Female	6	6	0.631
Tumor type^#^			
SRCC	12	2	
Non-SRCC	16	20	0.008^∗∗^
Tumor size			
>9.0 cm	6	0	
≤9.0 cm	21	21	0.021^∗^
Stage			
I-III	17	19	
IVA-IVB	11	3	0.045^∗^

^#^Tumor type is signet-ring cell carcinoma (SRCC), or it is not signet-ring cell carcinoma (non-SRCC). ^∗^*p* < 0.05, ^∗∗^*p* < 0.01, *χ*^2^ test.

## Data Availability

The datasets analyzed during the current study are publicly available from the following online databases: TCGA (https://tcga-data.nci.nih.gov/tcga/), TargetScan (http://www.targetscan.org/mamm_31/), and miRDB (http://mirdb.org/).
